# Infant feeding—a scoping review for Nordic Nutrition Recommendations 2023

**DOI:** 10.29219/fnr.v68.10456

**Published:** 2024-02-05

**Authors:** Agneta Hörnell, Hanna Lagström

**Affiliations:** 1 Department of Food, Nutrition and Culinary Science, Umeå University, Umeå, Sweden; 2 Department of Public Health, University of Turku and Turku University Hospital, Turku, Finland

**Keywords:** breastmilk, breastfeeding, breastfeeding statistics, Nordic and Baltic countries, infant formula, introduction of solid foods, health effects of infant feeding

## Abstract

The 2012 edition of the Nordic Nutrition Recommendations (NNR) included recommendations on breastfeeding, based on the most recent guidelines and recommendations from major national food and health authorities and organizations, systematic reviews, and some original research. For NNR 2023, the scope has been expanded and also includes formula feeding and the introduction of solid food. The main focus in this scoping review is on infants aged 0–12 months but also considers parts both before and beyond the first year, as the concept of ‘the first 1000 days’ emphasizes the importance of factors during pregnancy and the first 2 years of life for immediate and later health: physical as well as emotional and mental health. Breastmilk is the natural and sustainable way to feed an infant during the first months of life. Numerous studies have indicated immediate as well as long-term beneficial effects of breastfeeding on health for both the infant and the breastfeeding mother, and from a public health perspective, it is therefore important to protect, support, and promote breastfeeding. For full-term, normal weight infants, breastmilk is sufficient as the only form of nutrition for the first 6 months, except for vitamin D that needs to be given as supplement. The World Health Organization (WHO) and several other authoritative bodies therefore recommend exclusive breastfeeding during the first 6 months. Starting solids at about 6 months is necessary for both nutritional and developmental reasons. According to the European Food Safety Authority (EFSA) and the European Society for Paediatric Gastroenterology, Hepatology, and Nutrition (ESPGHAN), solid foods are safe to give from 4 months although exclusive breastfeeding until 6 months is the desirable goal. Breastfeeding can continue together with complementary foods as long as it is mutually desired by the mother and child. If breastfeeding is not enough or for some reason discontinued before the infant is 4 months of age, the infant should be fed infant formula, and, when possible, breastfeeding should be continued alongside the formula feeding. If the infant is 4 months or older, starting with solids together with continued breastfeeding and/or formula feeding is an option. Infant formulas have been developed for infants who are not breastfed or do not get enough breastmilk. Home-made formula should not be given.

## Popular scientific summary

- Most infants in the Nordic and Baltic countries are exclusively or partially breastfed from birth, and about 60–80% are still breastfed at 6 months.
- Breastmilk is sufficient as the only form of nutrition for the first 6 months for full-term, normal weight infants, but vitamin D needs to be given as a supplement.
- Both immediate and long-term health benefits of breastfeeding for the child and mother are well documented.
- When breastfeeding is not an option before 4 months of age, infant formula should be used, and home-made formula should not be given.
- Starting solid foods, including common allergens, at about 6 months is necessary for both nutritional and developmental reasons.
- A well-planned vegetarian or vegan diet, including relevant supplementation and fortified foods, can be adequate and appropriate for infants and children, but attention to several essential nutrients and monitoring growth is crucial.

The concept of the first 1000 days emphasizes the importance of factors during pregnancy (1, 2) and the first 2 years of life for immediate and later health: both physical (3) and emotional and mental health (4). These factors include parental environmental factors at conception as well as maternal nutrition during pregnancy and the way an infant is fed and cared for during the first 2 years of life. The concept is important as it guides actions and policies for improving both child and public health (5, 6). From a public health perspective, the World Health Organization (WHO) stresses the importance to protect, support, and promote breastfeeding (7), and breastmilk has even been called ‘personalised medicine for infants’ (3).

After giving birth, most mothers in the Nordic and Baltic countries breastfeed their infants. Most start with exclusive breastfeeding (Box 1), but this declines relatively quickly and less than half of the infants are still exclusively breastfed at 4 months. However, continued breastfeeding is common with about 60–80% still being breastfed at 6 months and about 30–60% at 12 months.

Box 1 Nomenclature used in the scoping review

**Table:** 

Infant feeding	Infant feeding means in this text primarily the first 12 months. In some parts, older children are also mentioned.
Exclusive breastfeeding	Breastmilk is the only food given. Infant may also receive vitamins, minerals, and medication. No water is given.
Total breastfeeding	Duration of breastfeeding, that is, the child´s age when breastfeeding is stopped.
Complementary foods^1^	Solid foods (depending on infant age the consistency can vary between purées, semi-solids, and finger foods) and other fluids than breastmilk.
Complementary feeding^1^	Breastfeeding in combination with solid foods. Water, formula, and other drinks can also be given.

1 WHO definition used (8). EFSA (9) and ESPGHAN (10) consider complementary feeding as ‘*Breastfeeding or infant formula in combination with solid foods*’.

## Methods

The present scoping review about infant feeding is a revision of the breastfeeding chapter in the Nordic Nutrition Recommendations (NNR) 2012 (11), which built on two systematic reviews focusing on health effects of breastfeeding and introduction of solids (12) and protein intake 0–18 years of age (13). This has been updated and extended with the most recent guidelines and recommendations from major national food and health authorities and results from three *de novo* systematic reviews performed within the NNR2023 project (14–16). The revision follows the protocol developed within the NNR2023 project (17) (Box 2). The main sources of evidence used in the review follow the eligibility criteria described by Christensen et al. (18).

Box 2 Background papers for Nordic Nutrition Recommendations 2023

- This paper is one of many scoping reviews commissioned as part of the Nordic Nutrition Recommendations 2023 (NNR2023) project (17)
- The papers are included in the extended NNR2023 report, but, for transparency, these scoping reviews are also published in Food & Nutrition Research
- The scoping reviews have been peer reviewed by independent experts in the research field according to the standard procedures of the journal
- The scoping reviews have also been subjected to public consultations (see report to be published by the NNR2023 project)
- The NNR2023 committee has served as the editorial board
- While these papers are a main fundament, the NNR2023 committee has the sole responsibility for setting dietary reference values in the NNR2023 project

The texts about health effects of breastfeeding described in NNR2012 (11) was updated using results from systematic reviews as well as evidence-based guidelines and recommendations; original research was assessed and included as references when needed. In addition to texts about human milk oligosaccharides (abbreviation HMOs used for both naturally occurring in breastmilk and the manufactured HMOs added to formula), formula feeding and complementary feeding were assessed and added as needed.

## Breastfeeding

### Recommendations related to breastfeeding

Breastmilk is a natural and sustainable part of early infant feeding (19), and the health benefits of breastfeeding for both the child and the breastfeeding mother are well documented (3) (see below).

Exclusive breastfeeding for 6 months has been recommended by the WHO for more than 20 years (7, 20). This is a strong recommendation that applies to all countries and populations regardless of economic status or developmental level. Exclusive breastfeeding entails that the infant is given no food or liquid other than breastmilk but, if necessary, can receive vitamins, minerals, and medications as well. Globally, most official bodies recommend exclusive breastfeeding the first 6 months or has it as a desirable goal (21), including the Nordic countries (11), Europe (22), the United States (23), Canada (24), and Australia (25).

From 6 months of age, the WHO recommends that breastfeeding continues as part of a healthy diversified diet throughout the first 2 years or beyond (7, 26). The wording of the previous Nordic recommendation for total breastfeeding is somewhat different, stating that breastmilk is recommended as part of the diet until 1 year or longer, as mutually desired by mother and child. The proportion of infants in the Nordic and Baltic countries being breastfed for at least 1 year has increased, and the latest statistics show that about 30–60% of 12 months old infants are breastfed (see also section 4.3).

Before the infant is 4 months of age, if breastfeeding is not enough, discontinued for some reason or never started, the infant should be fed infant formula (Table 1). When possible, breastfeeding should be continued alongside the formula feeding. If the infant is 4 months or older, starting with solids together with continued breastfeeding and/or formula feeding is an option.

**Table 1:** Outline of recommended foods and drinks at different ages

Age of child	Preferred food	Possible food	Required supplements	Comment
0–4 months	Breastmilk	Infant formula	Vitamin D from 1 week of age^1,2^	
4–6 months	Breastmilk	Infant formula Solid foods^3^	Vitamin D^1,2^	Honey and nitrate-rich foods should not be given during the first year (see section Introduction of solid foods).
6–12 months	Breastmilk Solid food^3^ Water	Infant formula Follow-on formula Gruel/cereal-based drinks	Vitamin D^1,2^	Follow-on formula is on the market, but the WHO/WHA has clearly stated that these are unnecessary products (WHA 39.28).
One-year onwards	Solid food^3^ Water	Breastmilk Infant formula Follow-on formula Gruel/cereal-based drinks	Vitamin D until (at least) 2 years of age^2,4^	In Sweden and Finland, gruel/cereal-based drinks are traditionally used but are not necessary parts of children’s diets. A local milk product is used in Iceland composed according to Codex for follow-on formula. Sugary drinks should not be given during the first 2 years. Rice drinks should be avoided during first 6 years.

WHO/WHA = World Health Organisation and World Health Assembly

1 Vitamin D should be given to all children from 1 to 2 weeks of age until (at least) 2 years of age (10, 22, 23).

2 In Finland, Iceland and Norway, vitamin D supplementation to formula-fed infants depend on amount of formula eaten (27–29).

3 Depending on infant age the consistency can vary between purées, semi-solids, and finger foods.

4 Children older than 2 years should continue with extra vitamin D if they do not eat fish or enriched foods, if they are not exposed to the sun during summer.

### Content of nutrients and other components in breastmilk

Human breastmilk is a multifaceted fluid containing water, protein, lipids, carbohydrates, vitamins, minerals as well as non-nutritive bioactive factors, such as hormones, growth factors, antibodies, HMOs, and bacteria with metabolites (30–33) (Table 2). Breastmilk gives the newborn essential nutrients in an efficiently absorbed combination (34). The contents of human breastmilk differ significantly from the milk from other mammals. When feeding breastmilk is not an option, parents should be given guidance to feed commercial infant formula to their infants.

**Table 2:** Nutritional content of breastmilk, infant formulas (on average), and cow’s whole milk

Nutrient/100 g^1^	Breastmilk	Infant formula^2^	Cow’s milk
Energy, kJ	273	284	265
Energy, kcal	65	68	63
Protein, g	1.3	1.3	3.0
Fat, g	3.5	3.3	3.1
SAFA, g	1.3	1.3	2.2
MUFA, g	1.4	1.4	0.7
PUFA, g	0.6	0.6	< 0.1
LLA, mg	337	519	45
ALA, mg	69	77	12
EPA, mg	4	4	0
DHA, mg	11	7	0
Carbohydrates, g	7.4	7.7	4.8
Lactose, g	7.4	6.7	4.8

1 Based on the national Food Composition Database in Finland (https://fineli.fi/fineli/fi/index).

2 Ready-To-Feed, average.

The macronutrient content (as percentage of energy, E%) of mature breastmilk from the individual woman is relatively stable for as long as breastfeeding continues, but the content of vitamins and minerals is influenced by the mother’s diet, and the fat composition varies depending on the mother’s diet and weight status (35–37). For example, the levels of long-chain polyunsaturated fatty acids are highly dependent on the mother’s intake of seafood, linoleic and linolenic acids, and dietary supplements (35, 36).

All infants and young children living at northern latitudes need vitamin D supplements. Sun exposure is insufficient to prevent rickets in the Nordic and Baltic countries, and breastmilk does not contain sufficient vitamin D for prevention even if the mother takes vitamin D supplements (38). If not given a vitamin D supplement, there is a rapid decrease in the serum concentration of 25-hydroxyvitamin D during the first weeks of life to the level usually seen in rickets (39). Supplements should be in the form of vitamin D drops or a multivitamin-mineral supplement, and 10 µg (400 IU) per day is recommended from 1 to 2 weeks of age until (at least) 2 years of age as discussed in the scoping review on vitamin D for NNR2023 (40). For infants fed formula during the first year of life, there are special recommendations regarding vitamin D-supplementation in some countries (see section Formula feeding).

The iron in breastmilk is highly bioavailable and easily absorbed by the infant, and when maternal iron status is good and umbilical cord clamping is delayed at birth (38), most full-term newborns have sufficient iron stored in their bodies for about the first 6 months of life. Some infants, for example, prematurely born, born small-for-gestational age, or with high growth velocity, may be at risk for iron deficiency prior to 6 months (9, 10, 25, 41). By age 6 months, most breastfed infants require an external source of iron and complementary foods should include foods with sufficiently high iron content (e.g. meat, egg, whole grain cereals and bread, lentils, beans, and nuts). Formula-fed infants should receive iron-fortified formula in addition to solid foods (10, 42).

Pregnant and breastfeeding mothers who eat a vegan or vegetarian diet should ensure that they have an adequate intake of vitamin B_12_, vitamin D, iodine, and the omega 3-fatty acid docosahexaenoic acid (DHA), either through supplements or enriched products, in order to avoid risk of deficiency and to ensure optimal intake in the fetus and infant. This would also be advisable for women with a mixed diet who have a low intake of fish and dairy products.

When the breastfed child of a vegan mother is no longer exclusively breastfed, or the mother’s nutritional status is uncertain, the child should receive a supplement containing vitamin B_12_ and iodine. When the child begins with solid foods, they should also be given DHA in the form of algae oil (vegetarian omega 3). The European Food Safety Authority (EFSA) suggests 100 mg/day as an adequate intake (AI) for children aged between 6 and 24 months and 250 mg from 2 to 18 years (43). See also section Introduction of solid foods; *Plant-based diets* below.

There are over 200 defined HMOs, that is, oligosaccharides, constituting the third largest component in human milk after lactose and lipids (44, 45). The total amount and composition of HMOs in the milk are unique for each woman and strongly depends on the maternal genetics (44, 46), as well as maternal and child characteristics such as maternal pre-pregnancy body mass index (BMI), gestational age, parity, and infant sex (45). Although HMOs are only minimally digested prebiotics, they have many important health-related biological functions for the infant (33). These include shaping the development of the gut microbiome by promoting the growth of specific microbes, preventing pathogen invasion and attachment, and altering immune cell responses, possibly also in other sites throughout the body and not only in the infant gut. HMOs may thus affect infants’ risks of infections, allergies, and inflammation (33, 47, 48).

### Prevalence of breastfeeding in the Nordic countries

The Nordic countries have relatively high breastfeeding rates. After giving birth, virtually all mothers start breastfeeding their infants and in the Nordic countries, 40–50% of infants are exclusively breastfed for 4 months (Table 3). Exclusive breastfeeding rates then declines relatively quickly, but breastfeeding is commonly continued together with the addition of solids and other fluids than breastmilk (i.e. complementary foods; see Box 1). About 60–80% of infants are still breastfed at 6 months and about 30–60% at 12 months. Breastfeeding rates are similar in the Baltic countries, with 50–70% of infants breastfed at 6 months.

**Table 3:** Breastfeeding statistics in the Nordic and Baltic countries

	Weeks			Months																			
	1		>2	1		1.5 (6 weeks)		2			3		4			5		6			8	9	12
	Excl	Any	Danish full brf	Excl	Any	Excl	Any	Excl	Danish full brf	Any	Excl	Any	Excl	Danish full brf	Any	Excl	Any	Excl	Danish full brf	Any	Any	Any	Any
Denmark^1^			85.4						67.0					57.9					13.4				
Finland^2^				58	93			54		88	53	86	50		85	26	88	9		77		75	58
Iceland^3^	63	82				65	88	62		88	59	80				40	74	20		68	63		39
Norway^4,5^	87	97		81	93			74					39		82	5		2		78		63	48
Sweden^6^	71.8	93.2						60.9		82.8			49.9		73.4			11.9		64.8	51.8		30
Estonia^7^												81.6								68.5			
Latvia^8^						40.0	88.1				29.7	72.4						16.1		53.7			27.4
Lithuania^9^	91.4^9^											68.4^10^								49.6^10^			

1 National statistics for children born in 2022 (*n* = 34 951; 59.3% of birth cohort included in the statistics) (51) Note: Full breastfeeding is in Denmark defined as only breastmilk + allowed water or similar + a maximum of 1 formula meal per week. In Denmark, no ‘exclusive breastfeeding’ is registered (breastmilk as well as vitamins, minerals, and possibly medicines), as this WHO definition is seen as less appropriate for Danish conditions where there are no health risks for the child associated with not being fully breastfed. 2 months data are collected for age >9 weeks and 4 months for >17 weeks.

2 Based on survey in 47 municipalities in mainland Finland October 2019, 3418 families participated with children aged 2 weeks to 12 months, response rate 22% (52). Statistics weighted to match cohort born 2018. The figures in the table are based on the previous day’s feeding.

3 Based on national statistics for children born in 2021 (*n* = 4,879). Data from primary health care centers’ regular check-ups in Iceland (53).

4 Based on national sample of 3000 children born 1–29 March 2018. Data collection at 6 months of age, included 2182 children (73%) (54). Data collection at 9 and 12 months included 1966 children (66% of those possible to contact) (55).

5 Exclusive breastfeeding rate at 5 months was taken at 5.5 months

6 Based on national statistics for children born in 2021 (*n* = 105,549) (56). Data are missing from 6 of 22 regions (=14% of children born 2021), National average is calculated using estimates based on most recent data from these regions (57).

7 Based on national statistics for children who became 1 year old during 2022 (*n* = 13 101) (58).

8 Based on national statistics for children born in 2022 (*n* = 17 494) (59).

9 Based on national statistics for children born in 2020 (*n* = 23,400) (60) (https://www.hi.lt/uploads/pdf/leidiniai/Statistikos/Gimimu/gimimai_2020.pdf).

10 Based on national statistics for children born in 2018 (*n* = 28,149) (61).

When comparing breastfeeding statistics between countries and within a country over time, it is necessary to consider both how exclusive breastfeeding is defined and how data collection is performed, whether data is based on national data or smaller samples but also participation rates. Lack of standardized definitions and data collection methods, as well as absence of agreements about at which ages statistics should be collected, makes comparisons difficult (49, 50). This problem is clearly seen in Table 3 showing the most recent breastfeeding statistics from the Nordic and Baltic countries.

Vaz et al. (50) describe breastfeeding rates and annual change in 51 high-income countries based on nationally representative samples from various sources collected between 1985 and 2019. They looked at indicators related to start of breastfeeding (hours after delivery), proportion ever being breastfed (even of short duration), exclusivity of breastfeeding at different ages, and proportion still being breastfed at 1 and 2 years (including estimates for different ages between 9 and 24 months). Their conclusion was that breastfeeding practices seemed to be improving in most of these high-income countries, although they still fell short of the WHO recommendations, with a median of only about half of infants still being breastfed (one quarter exclusively) at 6 months, and one third still breastfed at 12 months.

NNR2012 (11) included breastfeeding statistics for children born between 2004 and 2010 (depending on country). It identified that breastfeeding rates in the Nordic countries had increased a lot from the mid-1970s, but that both breastfeeding initiation and duration of exclusive breastfeeding had started to decrease in some of the countries. It was therefore stated as very important to further protect, promote, and support breastfeeding in all the Nordic countries. This seems to have had some positive effects (Table 3), but it is still imperative to improve the quality and regularity of breastfeeding statistics to plan appropriate actions and to follow up on their results.

Compared to the data in NNR2012 (11), both negative and positive changes can be seen in the most recent available data for children born in 2018 – 2022 (Table 3). Only Sweden and Iceland had data about breastfeeding during the first week of the infant’s life in both the time periods, and in both countries a decline could be seen from 97 to 93.7% and 98 to 83%, respectively. In Norway, the proportion of infants receiving any breastfeeding at 4 and 6 months declined somewhat among those born in 2018 compared with those born 2006 but remained the same at 9 and 12 months. However, the rate of exclusive breastfeeding at 5 months fell from 25 to 5%. Also in Sweden, a small declining trend could be seen for both exclusive and any breastfeeding at 4 months, while rates were relatively stable at 6 months, and the proportion still being breastfed at 12 months almost doubled from 16 to 28%. In Iceland, the proportion exclusively breastfed at 6 months had almost trebled from 8 to 22%, while any breastfeeding was relatively stable, slightly above 70%. Being breastfed at 12 months increased from 27 to 39%, and 24% were reported as still being breastfed at 18 months of age. Also in Finland, both the proportion exclusively breastfed and total duration increased substantially, with the proportion still breastfed at 9 months almost doubled from 39 to 75% and increased by 24 percentage points at 12 months: from 34 to 58%. In Denmark, breastfeeding rates during the first 6 months had increased.

The reasons for the changes in breastfeeding rates in the eight countries are not well studied. Possible reasons include changes in how long the mother and infant stay at the maternity ward and routines at the maternity wards, the information received during pregnancy, the duration of parental leave and its distribution between parents, the amount of support the parents receive both related to breastfeeding as such and child care more generally, and not least the competence about breastfeeding among staff within the health care system.

## Formula feeding

If the mother does not want to breastfeed at all or if there is a problem with breastfeeding before the infant is 4 months of age (e.g. if breastmilk is not enough for the infant despite skilled breastfeeding counseling, more intensive breastfeeding, or if breastfeeding must be discontinued for medical or other reasons), the child should be fed infant formula. If possible, breastfeeding should be continued alongside formula feeding, as even small amounts of breastmilk have benefits for the infant. If the infant is 4 months or older, starting with solids together with continued breastfeeding and/or formula feeding could also be an option.

Manufacturers of infant formula strive to mimic the average nutrient composition of breastmilk and also add other constituents of breastmilk such as probiotics and prebiotics (62). The composition and ingredients of infant formula and follow-on formula are standardized through EU regulation. The EU regulation of infant formula (63) required in 2016 increased levels of vitamin D in infant formula compared with earlier regulation. This resulted in changed recommendations about vitamin D supplementation during the first year of life for infants fed formula in some Nordic countries (27–29). The changed EU regulation (63) also led to changed recommendations in Norway and Iceland regarding the common practice of using cod liver oil. However, in 2019, an amendment to the EU regulation lowered the upper level of vitamin D to the previous level due to concerns regarding too high intake of vitamin D among formula-fed infants.

The EU regulation from 2016 (63) also required increased levels of DHA. There is, however, no recommendation level for arachidonic acid (ARA) in infant formulas, even though the synthesis of both DHA and ARA from essential fatty acids is low in the first months of life, and international expert groups have brought up the importance to add both DHA and ARA to infant formulas (64–66).

Infant formula has previously been much higher in protein than breastmilk, but the levels have been decreasing and are now closer to that of breastmilk (67) (Table 2). This change will decrease the difference in protein intake between infants fed breastmilk and those fed infant formula and thus probably also health outcomes related to protein intake (see section Health effects of infant feeding; *Overweight and obesity*).

Unmodified milk from cows or other mammals (e.g. goats) is not suitable for children under 1 year of age (except in small amounts in food from the end of the first year). The reasons are that unmodified milk may overload the kidneys due to the high protein content and that the low iron content may contribute to iron deficiency. In infant formula, the quantity and quality of protein and fats have been modified to replicate breastmilk as much as possible, and it also contains more lactose, vitamins, and minerals than cow’s milk. Owing to these modifications, infant formula (and follow-on formula from 6 months) is suitable for infants, unlike regular cow’s milk.

Plant-based infant formulas are at present not available in stores in the Nordic countries although it may be purchased online. Plant-based milk alternatives such as soy, oat, and almond drinks and home-made milk alternative mixtures should not be given to infants during the first year, except in small amounts mixed with other foods (10). Drinks based on rice should not be given during the first 6 years due to its high content of arsenic.

## Introduction of solid foods

### Recommendations related to the introduction of solid foods

As described above, exclusive breastfeeding is recommended for about 6 months by most official bodies globally (22), including EFSA (9) and the European Society for Paediatric Gastroenterology, Hepatology, and Nutrition (ESPGHAN) (10). The recommendations regarding when solids should start vary, however, between *at about 6 months* (most common) and *between 4 and 6 months* (21).

Guidelines generally agree that solid food should not be introduced before the age of 4 months (21), and if extra food is needed before 4 months, infant formula should be given. EFSA states that some infants show developmental skills to handle semi-solids between 3 and 4 months of age and finger foods from around 4 months (but most not until 5–7 months) but adds that ‘the fact that complementary foods can be introduced before 6 months does not imply that this is necessary or desirable’ (9). Indeed, also in high-income countries, exclusive breastfeeding for 6 months provides protection from gastrointestinal infections compared with breastfeeding and introduction of solids from 4 months (68). So, if the infant is healthy and the mother wants to continue exclusive breastfeeding, she should not be encouraged or pressured to start giving solid foods before 6 months of age, when gradual transition into a diversified diet is recommended (see also section *Timing* below).

Regardless of age at introduction of solid foods, a varied diet based on all food groups including iron-rich foods is recommended (8, 26, 68) (see also section *Plant-based diets*). Early inclusion of potentially allergenic foods (e.g. peanuts, egg, fish, and cereals) is also recommended (9, 26, 68–70) (see also section Health effects of infant feeding; *Atopy and Asthma*).

Some foods can cause health risks if given during the first year (with increasing risk the younger the infant): foods naturally high in nitrate (e.g. leafy vegetables such as spinach, beet blast, arugula; water from some private wells) can cause methemoglobinemia (71), and honey may contain the bacteria *Clostridium botulinum*, which can cause botulism (72). In addition, when peanuts are given, it should not be as whole pieces as this might cause choking.

### Priming for healthy and sustainable food habits

There is strong evidence for associations between maternal diet during pregnancy and lactation and children’s later food preferences, and if the mother has a healthy diet, there are good chances that the child will have it too (73, 74). However, when it comes to children’s later food habits, there are also many different potential mediators, moderators, confounders, and covariates related to both exposure and outcome that are not always included in studies (73). One very important factor to consider is the composition of the family diet in later childhood. Getting accustomed to new foods is not only about what the mother eats during pregnancy and lactation or even what the infant is given when starting complementary feeding. It is also very much about the family’s overall diet; if food is not served in the home, there is little chance for the child to secure it as a lasting accepted and preferred food. Here, the Nordic preschool system can play an important role as a large proportion of children from 1 to 6 years of age spend part of their day and eat some of their meals there.

Regardless of country, a relatively small proportion of populations eats according to recommendations. The intake of vegetables is usually much below recommendations in all age groups and genders, which is detrimental to public health. In addition, a major change in food habits, including a large increase in the amount of plant foods, has been suggested to both improve human health and to support environmental sustainability (75). Changing established food habits is difficult, and introducing healthy and sustainable food habits already in early childhood is therefore imperative. It has been suggested to start complementary feeding with the introduction of a variety of vegetables to increase the exposure to these foods (76). Unfortunately, many vegetables have bitter tastes and can therefore take a bit longer for the infant to accept (77).

At birth, infants have an inborn preference for the tastes sweet and umami and an inborn dislike for bitter and sour (77). A preference for sweetness matches well with the lactose-rich breastmilk, and sweet taste signals a higher energy content, which was advantageous in the beginning of evolution/humankind. However, it is less appropriate in the obesogenic society of today. Sugar should be avoided because it contains only calories and not the nutrients that infants need.

While a preference for salt is not inborn, it can develop early if salty foods are given (77, 78). Salt (sodium) should be avoided for infants due to their immature kidneys but also to avoid encouraging the development of a preference for salty taste. If children are given sugar and salt already from a young age, their preference for sugary and salty tastes will increase, and there is a strong evidence base associating a large intake of sugary and salty foods with clear negative health implications during the life course. Sugary drinks are, for instance, strongly associated with increased risk for overweight and obesity (79) and should not be given to children during the first 2 years (23). High salt intake has been shown to be associated with increased blood pressure already in infancy (80), and increased blood pressure is a major risk factor for stroke and cardiovascular disease in adulthood (81).

**Fig. 1:**
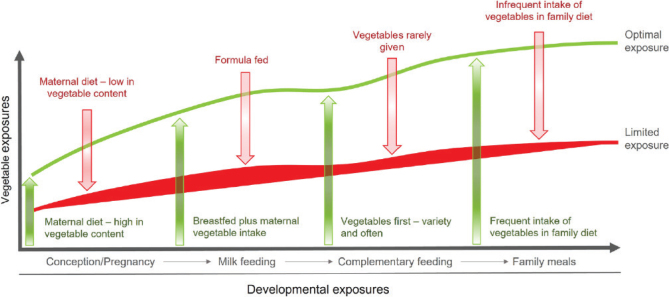
From: Nekitsing and Hetherington (76) republished under a Creative Commons license.

Bitter tastes are most likely disliked because it signals a risk of toxicity (77). When foods with bitter tastes, such as some vegetables, are first introduced, most infants react with reluctance to eat or even show distaste. There is a risk that parents take an initial dislike to heart and do not offer the unpopular food again. It is therefore crucial that parents are aware that these inborn preferences and dislikes exist and importantly can be modulated by early experiences, even as early as in utero (77). In their figure, Nekitsing and Hetherington (76) show how exposure to vegetables can work positively (green arrows) or negatively (red arrows) during four different (food) phases, from conception to the child eating family meals (Fig. 1). This could be an incentive for parents to improve their own diets, and many are also motivated to change when they expect a baby.

In many Nordic countries, foods with a relatively neutral (e.g. potatoes) or sweet taste (e.g. carrots) have traditionally been used as the first foods when complementary feeding starts. Encouraging parents to introduce a variety of foods with ‘more demanding’ sensory qualities can be positive for later acceptance. A recent Swedish randomized controlled trial (RCT) (82) showed that starting complementary feeding with a systematic introduction of inherently sour or bitter-tasting Nordic fruits (apple), berries (raspberry, lingonberry, cranberry), roots (turnip, white radish), and vegetables (cauliflower) resulted in overall higher intakes of fruit and vegetables at 9, 12, and 18 months of age.

Although the child should be exposed to various vegetables and fruits in the 6–12 months period to increase acceptance and secure future food habits, fruits and vegetables have a low energy content and also often a low iron content. Infants and young children have small stomachs, and enough space should be ensured for energy and iron rich foods for the infant to meet energy and nutrient needs. This is especially important if a vegetarian or vegan diet is pursued (see also section *Plant-based diets* below).

### Timing

Children’s food preferences start already in the womb with the amniotic fluid flavored by maternal diet, followed (for the breastfed infant) by a chemosensory continuity in the breastmilk (83). Breastfed infants therefore experience a large variation of flavors during feeding, partly due to the mother’s dietary habits and partly because the content of lactose and fat in the breastmilk changes during a breastfeed, which gives even larger variation for the infant. In contrast, formula-fed infants get the same flavor sensation every time, unless there is a change of brands.

It is sometimes stated that it is important to start introducing semi-solids no later than 4 months, sometimes even before 4 months, often referring to a study by Mennella et al. (84). This was an RCT testing if acceptance to bitter tastes, which usually are disliked, could be influenced by the age at introduction. They saw that switching infants fed on cow’s milk formulas to protein hydrolysate formula (PHF), which is very bitter, before 3.5 months was readily accepted, while a switch at 3.5 months was less accepted, although longer exposure increased acceptance. In an earlier study, they had seen that the introduction of PHF at 4 months or later was difficult (85), and they therefore concluded that their results suggest that the ‘window’ for early acceptance and long-term influences is beginning to close at about 3.5 months (84). These results cannot, however, be interpreted as evidence of a window of opportunity for the introduction of solids in breastfed infants.

It has been shown that breastfed infants faster get accustomed to and accept foods that the mother has eaten during pregnancy and lactation than noneaten foods and also faster than formula-fed infants, who only get the flavor experience of their mother’s diet from the amniotic fluid. For the individual family, the importance of accustoming infants to different tastes could be one aspect to consider when deciding at what time point they should introduce complementary foods to their infant. In Finland and Iceland, the introduction of (semi-) solid foods is recommended already at 4 months of age for formula-fed infants who do not get breastmilk at all, to ensure that the infant gets the sensory sensation of different tastes (86, 87). In Sweden, since 2011, parents are told that, regardless of feeding method, infants that seem interested in the family foods can be given ‘tiny tastes’ (≤1 mL) from 4 months (17 weeks) (72). The amounts of these tastes should not increase until about 6 months of age as the intention is sensory sensations, not increased nutrient intake, and is therefore seen as not competing with breastfeeding or formula feeding. However, a recent study showed that the earlier infants were given tiny tastes the earlier they ate larger amounts of solid foods and the shorter the breastfeeding duration (88).

There are several developmental milestones that signal that an infant is physically ready to start the process of changing from milk feeding to family foods (9). Some of these milestones are easy to detect, such as the ability to sit up without support and holding up the head (decreasing the risk for choking), the extrusion reflex of the tongue that prepared the infant for suckling decreases (making it easier for the infant to handle solids and not accidently spit it out), increased reaching and trying to pick up things, and an interest in putting those into the mouth. Some of these milestones can occur already at 3–4 months of age but more commonly at 5–7 months. A child showing these signs of physical maturation before 6 months of age does however not imply that there is a nutritional need for complementary feeding to start.

When discussing the timely introduction of complementary foods, the subject of *sensitive* and *critical periods* often comes up. It is important to differentiate between these. During a *sensitive period*, it is easier to learn a new behavior; after that, learning is still possible but can be more difficult (89). In contrast, *critical periods* are more of a *window of opportunity*, during which the exposure *must* take place for learning to occur (or be very much more difficult). Learning to eat lumpy foods (i.e. foods that need to be chewed) seems, for instance, to have a critical period around 6–9 months of age, and if these foods are introduced later, there is a greater risk for later feeding problems (90). When it comes to taste preferences among children, learning to like new tastes seems to be more about sensitive periods, that is, it is never too late to learn to accept new foods (89).

Mennella et al. (84) describe the sensitive periods as *‘age-related changes in functional plasticity for human flavour programming’*, emphasizing that the early accustoming to the maternal diet through amniotic fluid and breastmilk works as a biological adaptation to the diet the infant will meet when complementary feeding starts. Phenotypic plasticity during development is also described by Burggren and Mueller (91). However, their description of sensitive/critical periods is more complex than the traditional definition, which has relatively distinct starting and stopping times. Burggren´s and Mueller’s definition includes a three-dimensional construct where time during development, dose (e.g. amount eaten), and the resulting phenotypic modification are all considered. Intuitively, this more multifaceted thinking goes well with the notion that although it is harder to learn new food habits when older, humans are able to learn new flavor preferences throughout their lifespan and when necessary, even change their whole diet.

Starting solids before 4 months is discouraged mainly because neither renal nor gastrointestinal function is mature enough before that age (9). The risk for choking is also greater. On the other hand, delaying the start of solids until after 6 months may increase the risk of iron deficiency. However, few infants in the western world are exclusively breastfed beyond 6 months, including the Nordic and Baltic countries (Table 3), so there is a scarcity of data to enable quantification of the risk. Indeed, EFSA (9) concludes that the biggest risk factors for iron deficiency are early umbilical cord clamping, being born preterm or with a low birth weight. It is, however, recommended that foods rich in iron should be among the first to be given to exclusively breastfed infants when the accustoming to solids starts.

### How to introduce foods – parental-led or baby-led?

Children are evolutionary prepared to learn and adapt from cues in their environment. How fast an infant accepts new foods depends on several factors, for example, type of milk-feeding, level of neophobia, sensory sensitivity, and age at introduction, but the way new foods are introduced also affects the level of acceptance (92). Humans are social beings, and not only physiological but also psychological and social dimensions are important for how tastes, foods, and meals are experienced. There are a range of primary mechanisms that can be used to achieve increased acceptance for new foods (73), for example, associative conditioning (giving a new food together with something already familiar), repeated exposure (whereby the new food becomes more and more familiar), variety exposure (the larger the variety of foods the child has already accepted, the easier it is to introduce new similar foods), and social modeling (eating together with others, especially other children who already like the novel food, increases acceptance).

In most guideline documents on infant feeding, responsive feeding is recommended (21). This includes eating in a pleasant environment where the parent focuses on the infant through talk and eye-to-eye-contact. It is also commonly recommended to avoid using food as consolation or reward; hunger and satiety cues should guide the feeding. Parents should also avoid forcing children to eat and instead encourage self-feeding and self-regulation.

The term *baby-led weaning* has been used, and it usually includes that spoon-feeding by the parents is avoided and instead infants take the food themselves when solid food is introduced at around 6 months (93). However, the systematic review by D’Auria et al. found that there is no clear definition of the term, and most studies include varied combinations of spoon-feeding and self-feeding, making it difficult to evaluate the possible advantages and disadvantages of strictly avoiding spoon-feeding.

### Plant-based diets

Regardless of what type of diet parents choose to give their children, it is important that they are knowledgeable so they can manage to plan and cook an adequate diet to support their child’s immediate and future health. Vegan and vegetarian diets may provide health benefits for both prevention and treatment of certain diseases, including cancer, cardiovascular disease, and diabetes among adults (10, 94). Due to the ongoing global transition to decreased meat intake and a more plant-based diet (75), there is an increased public health interest in the adequacy of vegetarian and vegan diets. Naturally nutrient-dense foods are in general preferable, but as certain nutrients are difficult or impossible to eat in sufficient amounts without intake of foods of animal origin, an increase in the availability of fortified foods and supplements has made it easier to plan and achieve an adequate vegan or vegetarian diet.

The view regarding whether a vegetarian or vegan diet can be given to children has changed radically during the last decades (95). A summary of existing guidelines from a range of health or government organizations from, for example, the USA, Europe, New Zealand, and Australia concludes that *well-planned* vegetarian and vegan diets, including relevant supplementation and fortified foods, are nutritionally adequate and appropriate for all stages of the life cycle, including pregnancy, lactation, infancy, and childhood (21). A large German study on dietary intake and health among vegetarian, vegan, and omnivore children aged 1–3 years concluded that energy intake was the same and normal growth achieved in all three groups (96). Regarding micronutrients and fatty acids, the results were more diverse, although showing that the vegan and vegetarian diets (including supplements and fortified foods) provided sufficient amounts of most micronutrients and had a better fat quality than the omnivore diet (97). The intake of some nutrients (vitamin E, vitamin B_1,_ folate, magnesium, iron) and fat quality were most favorable for the vegan children and least for the omnivores (vegetarians in-between), while the opposite was true for others (vitamin B_2_, calcium, iodine, and DHA). The study identified some (potentially) critical nutrients: vitamin D, iodine, and DHA among children regardless of diet and in addition vitamin B_2_, B_12_, calcium, and iron for children on vegan or vegetarian diets.

The importance of careful planning and inclusion of supplementation and fortified foods is crucial when implementing a vegan or vegetarian diet to children as they need a more nutrient-dense diet than adults to ensure normal growth and development. The more restricted a diet is, that is, the fewer food types chosen, the more important the nutrient content of the foods eaten and the more knowledge required for planning the diet. When planning meals, it is also important to consider the combination of foods that may affect the uptake of different nutrients, for example, phytates decrease iron and zinc absorption, while vitamin C increases uptake. It is also necessary to know when supplements or enriched food products are needed to meet nutritional requirements as the consequences of low intake of some nutrients can be severe and irreversible. In fact, although ESPGHAN is among the organizations stating that a well-planned vegan diet is safe for children, they also state that ‘*Vegan diets should only be used under appropriate medical or dietetic supervision*’ (10).

A healthy and balanced vegetarian or vegan diet can be composed of legumes (beans, lentils, peas), whole-grain cereals, vegetables, fruit, berries, nuts, seeds, plant oils, plant-based drinks, and low-fat dairy products and eggs if included in the diet (94). Nutrients that require extra attention are vitamin B_12_, vitamin D, iron, iodine, selenium, zinc, calcium, omega-3 (DHA), and protein. Good sources for some of these minerals, for example, selenium, may differ due to the amount in the soil where the plants were grown. For B_12_ and several of the other mentioned nutrients, supplements or fortified foods are necessary to meet nutritional requirements. A *de novo* systematic review was performed within the NNR2023 project to assess whether the intake of vitamin B_12_ from habitual diet or supplements is enough to cover the needs for groups most susceptible to vitamin B_12_ deficiency; including pregnant and lactating women as well as infants, especially when a vegan or vegetarian diet is followed (98). Due to a lack of evidence, no conclusion could be made. While waiting for more research to be performed, pregnant and lactating women need to ensure an adequate intake of vitamin B_12_ from supplements, foods enriched with sufficient amounts of vitamin B_12_ or injections.

It is also necessary to ensure that children eating a plant-based diet can manage to eat enough food to sustain adequate growth and development. The composition of plant-based diets usually results in a high fiber and relatively low energy content. This can make it difficult for children to eat enough to meet their energy needs, especially for young children, as the volume of the food required can get too large. Parents therefore need to make sure that children follow their growth curves. The optimal level of dietary fiber in the diets of children is unknown, but EFSA suggests that from 1 year of age, a daily intake of 2 g of fiber/MJ is an adequate amount for achieving normal bowel movements and avoid constipation due to too low intake (99). The most recent dietary guidelines for Americans indicate a higher level, with a suggested 14 g of fiber/1000 kcal for children aged 12–23 months (approximately 3.3 g/MJ) (23). A de novo systematic review within the NNR2023 project found no clear associations between a high fiber intake and growth among children 0–5 years of age and found no studies on high fiber intake and its relation to iron status (16).

If the family eats a vegetarian or vegan diet, continued breastfeeding after 1 year of age or longer is recommended (94). For vegan families where the infant requires more milk than breastmilk, the options are limited. At present, there are no vegan infant formulas for sale in stores in the Nordic countries although it may be purchased online.

A growing assortment of various plant-based products and processed foods in recent years has made it easier to achieve a healthy plant-based diet also for young children, and many of these foods are fortified with micronutrients that would otherwise be nonexistent, with low concentration or low bioavailability. It is important to be aware that the levels of fortification of similar foods can vary manifold (100). Parents therefore need to read the information on packages carefully, so their children do not exceed tolerable upper limits of nutrients (e.g. iodine) through eating a combination of fortified foods with high levels of fortification, especially if they also eat supplements.

Regulations regarding the fortification of different foods vary within Europe (101). For instance, in Sweden, it is since 2018 legally required for producers to add vitamin D in most milk-based products and their nonmilk counterparts (based on soy, oat, rice, and almond), including organic products (102). In many, but not all, plant-based alternatives for milk and yogurts sold in Sweden, other vitamins and minerals are also added, for example, vitamin B_12_, iron, calcium, etc., but in organic products only legally required fortification is allowed (i.e. vitamin D). It is also important to realize that processed foods are not necessarily healthy just because they are plant based. Note that rice drinks (and rice cakes) should not be given to children below 6 years of age due to the content of arsenic (10).

Adequate intakes of vitamin B_12_ by pregnant and lactating women are crucial to avoid irreversible, negative effects on the fetus and infant (10). The main natural sources of vitamin B_12_ are foods of animal origin. Small amounts of B_12_ analogues can be found in seaweed and fermented vegetables, but these are not bioavailable for humans and are therefore not a source of B_12_ (94, 103). In addition, seaweed (and kelp) should be used with great caution by pregnant and breastfeeding women and infants due to the high variability in iodine content.

## Health effects of infant feeding

Many nutrients and biologically active substances are found in breastmilk, including vitamins, minerals, fatty acids, and immune factors, and many of these have proven positive effects on health. Nutrients in human milk have multiple functions related to optimal growth, development, and physiological functions. Deficiencies during the first 2 years may cause permanent damage. Numerous studies have indicated long-term beneficial effects of breastfeeding on health for both the infant (Table 4) and the breastfeeding mother (3). Indeed, Victora et al. even characterize breastmilk as a personalized medicine for infants.

**Table 4:** Evidence for health effects associated with infant feeding.

Outcome	Evidence	References
Infections	Convincing evidence that breastfeeding has a protective effect against overall infections, acute otitis media, gastrointestinal infections, and respiratory tract infections in childhood.	(3, 68, 104, 105)
Overweight and obesity	Probable evidence that longer duration of breastfeeding, exclusive or any, have a protective effect against overweight and obesity in childhood and adolescence, as well as in the general population. Probable evidence that high protein intake in during the first 18 months cause higher BMI later in childhood.	(3, 15, 93, 94, 95, 96, 97, 98, 99, 100, 101) (3, 14, 106–114)
Celiac disease	Moderate to high level of confidence that breastfeeding during or after introduction of gluten has no protective effect against celiac disease in early childhood. The amount of gluten at introduction may have an effect on the risk for celiac disease, but whether it prevents celiac disease or merely delay the start is unclear. The evidence is insufficient to conclude which age is best for the introduction of gluten; therefore the recommendation is to start giving gluten containing foods when complementary feeding is started.	(9, 10, 101) (9, 10, 112)
Type 1 and 2 diabetes mellitus (T1DM and T2DM)	Limited to moderate evidence that any breastfeeding and longer duration of any or exclusive breastfeeding protects against T1DM. Moderate to strong evidence that introduction of gluten, cow’s milk, and fruit increase the risk for T1DM Probable evidence that any breastfeeding has a protective effect against T2DM. The evidence for a stronger protective effect for longer duration of breastfeeding is limited but suggestive.	(3, 10, 102, 103) (3, 10, 115, 116)
Cardiovascular disease	Probable evidence that breastfeeding has a small reductive effect on blood pressure and lower triglycerides in childhood and adolescence, as well as a small reduction in blood cholesterol levels in adulthood for the breastfed infant.	(12, 104, 105, 106, 107, 108, 109, 110, 111) (12, 115, 117–123)
Brain development	Prolonged breastfeeding has been related to improved performance in intelligence tests and a positive effect of breastfeeding on cognition and visual acuity	(3, 108, 109, 112, 113, 114, 115, 116) (3, 106, 120, 122, 124–128)
Atopy and asthma	The evidence is strong that the previous recommendation to avoid allergenic foods during at least the first 12 months increase the risk for atopy and asthma. There is moderate evidence that introduction of well-cooked egg, and peanuts in populations with a high degree of peanut allergy, as part of complementary feeding but not before 4 months of age, may decrease the risk of atopy and asthma. There is low evidence that avoiding supplementation with regular cow’s milk formula during the first week of life in breastfed infants decrease the risk for atopy and asthma. No conclusions can be drawn for preventive effects of breastfeeding in itself on the risk for atopic diseases or asthma in children.	(12, 57, 58, 113, 117, 118, 119, 120) (12, 69, 70, 124, 129–132)

The positive effects seen for breastfeeding depend to a considerable degree on the breastmilk itself and its unique composition (133). The effects may also depend on the physical closeness during the act of breastfeeding or on other associated factors. An important factor is also what nonbreastfed or partly breastfed infants are fed instead of breastmilk.

Interpreting the results of different studies is difficult because the definition of breastfeeding varies between studies and the methodology used to assess breastfeeding is often unclear. The impact of breastfeeding and the level of evidence often varies for different health-related outcomes. Research in this area has been active not least because of the high interest in the programming effect that diet can have on future health (133).

### Infections

There is convincing evidence that breastfeeding has a protective effect against overall infections, acute otitis media, gastrointestinal infections, and respiratory tract infections also in high-income countries (3). Breastmilk contains many protective factors that might exert long-term health benefits, and the immunological protection against otitis media appears to last for some years after cessation of breastfeeding (105). The magnitude of the effect varies depending on the specific outcome and the exclusiveness of breastfeeding. Systematic reviews cited in Victora et al. (3) showed that longer and/or more breastfeeding (e.g. ever vs. never, exclusive vs. not exclusive, long duration vs. shorter) resulted in approximately 30% risk reduction for otitis media and lower respiratory tract infections during the first 2 years, and for gastroenteritis during the first 5 years. Earlier, it has also been stated that exclusive (or predominant) breastfeeding for 6 months compared to 3–4 months gives better protection against mild gastrointestinal infections (68) and that any breastfeeding reduces the risk of diarrhea (104).

### Overweight and obesity

Longer duration of breastfeeding, exclusive or any, has a probable protective effect against overweight and obesity in childhood and adolescence as well as reduces the occurrence of overweight and obesity in the general population by about 30% (3, 106–108). Mechanisms linking breastfeeding to lower incidence of early childhood obesity are still mostly unclear, but several plausible pathways have been suggested, for example, the changing nutrient composition of breastmilk during a breastfeed improves recognition of satiety cues improving self-regulation of intake (109), the lower protein content of breastmilk compared with infant formula (14), protective effects by non-nutrient bioactive components in breastmilk (110, 111), and epigenetic effects and programming connected with breastmilk (113).

There is probable evidence for a cause-and-effect association between higher total protein intake during the first 18 months of age and higher BMI later in childhood as shown by a *de novo* systematic review within the NNR2023 project by Arnesen et al. (14). The meta-analysis of five cohort studies showed that for each additional E% of protein, BMI (kg/m^2^) increased with 0.06 (95% confidence interval [CI]: 0.03–0.1) BMI units. This seemed to be driven by the proportion of protein of animal origin, as no associations were found between intake of plant-based proteins and later BMI or body fat (albeit based on few studies). Despite the decreasing levels of protein in infant formula to better match the content in breastmilk, it will take time before this may be reflected in the prevalence of overweight and obesity in the population.

The WHO growth charts from 2006 (114) are based on breastfed infants and give a good picture of normal growth. Use of the WHO growth charts for children is therefore recommended (112). Using growth charts based on children born when the breastfeeding rates were lower than nowadays (like in the 1970s) may lead to misinterpretations by health care staff, as infant formulas at that point in time contained more protein than modern formulas. This could cause erroneous advice about the need for additional food to breastfed infants, inadvertently increasing the risk for shortened breastfeeding duration and associated long-term health effects.

### Celiac disease

Previously, it was thought that breastfeeding could protect from celiac disease, especially if the infant was still breastfed when gluten was introduced. However, a later meta-analysis including two large RCTs has not confirmed this but instead suggested that the later introduction of gluten led to a delay of the start of celiac disease rather than prevented it (134). Therefore, both EFSA (9) and ESPGHAN (10) recommend introducing gluten-containing foods during the first 12 months when other complementary foods are introduced. However, they recommended that the amount of gluten should be kept low during introduction and large quantities avoided onwards throughout infancy.

### Diabetes mellitus, type 1 and 2

Whether different aspects of infant feeding including breastfeeding and gluten intake could be involved in the development of type 1 diabetes mellitus (T1DM) has been studied for many years. ESPGHAN concluded in 2017 that breastfeeding at the time of introduction of gluten did not influence the risk of T1DM (10). Very early introduction of gluten (before 3 months) was associated with a higher risk to develop diabetes among children with increased risk. However, starting with gluten after 3 months of age did not increase the risk, so the recommendation to introduce gluten when other foods are introduced sometime between 4 and 12 months of age was not changed. Later evidence suggests, however, limited to moderate evidence for an increased risk of T1DM for less or no breastfeeding (135) and moderate to high certainty for an increased risk with shorter breastfeeding (exclusive or any) and earlier introduction to gluten, cow’s milk, or fruit (116). There is probable evidence that any breastfeeding has a protective effect on type 2 diabetes mellitus (T2DM) (3), which might be connected to the association with the risk for overweight and obesity.

### Cardiovascular disease

The influence of breastfeeding on the risk of cardiovascular disease is unclear, but there is probable evidence of small but significant effects on the reduction of blood pressure levels and serum cholesterol levels in adulthood for the breastfed child (12). The association with blood pressure levels in childhood is, however, not clear as there are studies not showing this association (115). One possible influencing factor could be that a higher intake of n-3 fatty acids by breastmilk might affect the elasticity of the blood vessels. A protective effect against high blood pressure has been seen for the breastfeeding mother (136). The lower cholesterol level in adulthood found among those who were breastfed in infancy might be the result of the metabolic effects of constituents in breastmilk such as cholesterol and n-3 fatty acids (118). Breastfeeding has been reported to have a protective effect on triglyceride levels in childhood and adolescence (123). Indeed, the increased risk of overweight and obesity in later life for nonbreastfed infants compared with breastfed infants is in itself a risk factor for cardiovascular diseases (121).

### Brain development

Breastfeeding has been related to improved performance in intelligence tests, and a positive effect of breastfeeding on cognition was observed in a systematic review (3, 125). The favorable effect of breastfeeding on the healthy neurological development of the infant might be caused by the high content of DHA in breastmilk because this fatty acid is present in high amounts in nerve cell membranes.

However, the interpretation of studies on the association between breastfeeding and neurological development is complicated because the outcome is not only influenced by whether the child is breastfed or not, nor only by what children are fed instead of breastmilk and the exposure that this gives. The interaction between the mother/caregiver and the infant in the feeding situation is also important. It has also been shown that genes might affect the associations between breastfeeding and outcomes such as cognition (124), and this suggests that genetic aspects should be included in future studies.

Positive results from the PROBIT study in Belarus that compared control areas to intervention areas in which breastfeeding was promoted also provide quite strong support for positive associations between breastfeeding and neurological development (126). The nonresults in another paper from the same group (127) can probably be explained by the fact that this latter paper compared children who were exclusively breastfed for 3 or 6 months from both the intervention and control areas.

Other studies have also shown beneficial associations for breastfeeding with cognitive measures in children (120, 122). Oddy and coworkers concluded that although the effect sizes were small, any breastfeeding for 4 months or longer was associated with improved neurological outcomes in children aged 1 to 3 years after adjustment for multiple confounders (120). In a Danish national birth cohort, breastfeeding duration of 1 month or shorter compared with longer periods was associated with lower IQ even after adjustment for maternal IQ and other factors (122). However, the authors stated that there might be other pre- and postnatal factors that influence child IQ not taken into account. One prenatal factor that may influence cognitive development in children is supplementation with omega-3 fatty acids during pregnancy although a recent systematic review concluded that the evidence was limited (128).

### Atopy and asthma

The recommendations related to infant feeding and development of atopic diseases and asthma have changed over time. Previous advice on allergy prevention included exclusive breastfeeding for 6 months and avoidance of allergens through the delayed introduction of potentially allergenic foods to the infant’s diet until after 12 months or longer and sometimes also avoidance for the woman during pregnancy and lactation. Immunomodulatory qualities of breastmilk were thought to prevent conditions such as asthma, especially if a family history of atopy was present. The systematic literature review performed for NNR2012 (12) found the existing scientific evidence on the associations between avoidance of allergenic foods during pregnancy and lactation, duration of exclusive breastfeeding, and age at introduction of complementary feeding on later development of atopic diseases and asthma limited and contradictive, and therefore no specific recommendations regarding introduction of allergenic foods were given.

Since then, results from a number of new RCTs (e.g. 129–132) and systematic reviews (e.g. (117, 119) have been published. The evidence has been strongest for decreasing prevalence for allergies against peanuts and egg with early introduction. This has resulted in updated guidelines from the European Academy of Allergy and Clinical Immunology (EAACI) (70), focusing on preventing food allergy during the first 5 years. The EAACI-guidelines recommend that when solid foods are introduced, *well-cooked* eggs should be among the foods given, and in populations with high prevalence of peanut allergy, peanuts (in an age-appropriate form) should also be among the served food. It is also advised against giving cow’s milk infant formula during the first week of life to breastfed infants, but the level of evidence is low. Joint guidelines from USA and Canada not only have similar messages but also include a suitable age stating that to prevent peanut and egg allergy, these foods should be introduced around 6 months but not before 4 months (69).

After the publication of these guidelines (69, 70), two new RCTs have been published suggesting that the introduction of peanuts already at 3 months is even more efficient in decreasing the prevalence of allergy, without negative effects on breastfeeding (130, 132). The recommendation of exclusive breastfeeding for the first 6 months is therefore put into question. However, as the research teams encouraged their participants to breastfeed, it is not sure that breastfeeding would remain unaffected in the general public if a very early introduction of solids is recommended to decrease the risk for allergies. Indeed, a Swedish study saw that a change in the infant feeding recommendations in 2011, suggesting the introduction of tiny tastes (<1 mL) to interested infants already from 4 months, but without increasing the amounts until about 6 months of age, inadvertently led to shorter breastfeeding duration (88). It seems prudent to suggest that the introduction of allergenic foods should start when complementary feeding is started but not before 4 months.

Genes might have modifying effects also on the associations between breastfeeding and outcomes such as allergies (124), and this suggests that genetic aspects should be included in future studies. Very little is known about the possibilities of active prevention of allergies and asthma by adding specific food components or dietary supplements such as n-3 fatty acids, pre- and probiotics, or vitamins to the diets of pregnant or lactating women or to the diets of infants. Any positive effect of such additions remains to be shown.
